# Selection and assembly of indigenous bacteria and methanogens from spent metalworking fluids and their potential as a starting culture in a fluidized bed reactor

**DOI:** 10.1111/1751-7915.13448

**Published:** 2019-07-21

**Authors:** Ioannis Vyrides, Damian W. Rivett, Kenneth D. Bruce, Andrew K. Lilley

**Affiliations:** ^1^ Molecular Microbiology Research Laboratory Pharmaceutical Science Research Division King's College London 150 Stamford Street, Franklin‐Wilkins Building London SE1 9NH UK; ^2^ Division of Biology and Conservation Ecology School of Science and the Environment Manchester Metropolitan University Manchester UK; ^3^ Present address: Department of Environmental Science and Technology Cyprus University of Technology 30 Archbishop Kyprianos 3036 Lemesos Cyprus

## Abstract

Waste metalworking fluids (MWFs) are highly biocidal resulting in real difficulties in the, otherwise favoured, bioremediation of these high chemical oxygen deman (COD) wastes anaerobically in bioreactors. We have shown, as a proof of concept, that it is possible to establish an anaerobic starter culture using strains isolated from spent MWFs which are capable of reducing COD or, most significantly, methanogenesis in this biocidal waste stream. Bacterial strains (*n *=* *99) and archaeal methanogens (*n *=* *28) were isolated from spent MWFs. The most common bacterial strains were *Clostridium* species (*n *=* *45). All methanogens were identified as *Methanosarcina mazei*. Using a random partitions design (RPD) mesocosm experiment, we found that bacterial diversity and species–species interactions had significant effects on COD reduction but that bacterial composition did not. The RPD study showed similar effects on methanogenesis, except that composition was also significant. We identified bacterial species with positive and negative effects on methane production. A consortium of 16 bacterial species and three methanogens was used to initiate a fluidized bed bioreactor (FBR), in batch mode. COD reduction and methane production were variable, and the reactor was oscillated between continuous and batch feeds. In both microcosm and FBR experiments, periodic inconsistencies in bacterial reduction in fermentative products to formic and acetic acids were identified as a key issue.

## Introduction

Metalworking fluids (MWFs) are oil‐ and water‐based fluids, employed during the machining and shaping of metals to provide cooling, lubrication and inhibit corrosion. Waste MWFs are a major global pollutant whose disposal is increasingly costly to metal engineering industries (Cheng *et al*., 2005).

It has become increasingly popular to treat high chemical oxygen deman (COD) wastes anaerobically in bioreactors. Anaerobic bioreactors have the advantage that they generate less end‐point biomass, cope well with high organic loading, require fewer energy inputs and generate biogas (methane) which is valued as a clean and sustainable energy source. However, waste MWFs have proven difficult to introduce into anaerobic bioreactors because of the strong biocidal components included to protect them in use. Numerous components are rarely disclosed due to commercial sensitivity; however, more than 300 different substances are known to be used in individual fluids (Rabenstein *et al*., 2009). The main known compounds in MWF that have antimicrobial properties are alkaloamines, for example monoethanolamine (MEA), triethanolamine (TEA) and benzotriazole (BTA) (Jagadevan *et al*., 2013). MEA and TEA can be toxic to bacterial cell membranes due to their surface‐active properties, whereas BTA is a commonly used biocide in metalworking fluids and is also poorly biodegraded (Jagadevan *et al*., 2013). MWF has a high COD in the range of approximately 10–100 g l^−1^ (Amin *et al*. 2017), whereas nitrogen content, phosphorous content and pH are in the range of 450 ± 50 mg l^−1^, 70 ± 15 mg l^−1^ and 9 ± 0.5 respectively (Ławniczak and Marecik, 2019).

To date, several studies have investigated the microbial composition of MWFs (Gilbert *et al*., 2010). These studies were focused on the presence of aerobic bacteria. Few studies have examined MWF for the presence of anaerobic bacteria (Van der Gast *et al*., 2001; Bakalova *et al*., 2007; Di Maiuta *et al*. 2017). Recently, Di Maiuta *et al*. (2017) used parallel ribosomal gene tag sequencing to profile microbes from industry‐based liquid in water‐miscible MWF samples. Archaeal DNA was only found in two of 78 samples analysed. All reads were classified into the genus *Methanobrevibacter*. Interestingly, they were no longer detectable upon treatment. This suggested that only detectable DNA and/or compromised cells were present, rather than active microbial cells. According to Di Maiuta *et al*. (2017), *Methanobrevibacter smithii* was described as colonizer of the human gut system; thus, they probably had their origin from human sources. Methanogenic archaea are key to the successful operation of anaerobic bioreactors. These archaea, however, are known to be sensitive to chemical challenges, and the introduction of waste MWFs into anaerobic digesters has been shown to have highly deleterious effects on the production of methane (Perez *et al*., 2006). Teli *et al*. (2015) used a biochemical methane potential (BMP) test and found a minor production of methane over a period of approximately 150 days when starting with 0.5% of fresh MWF. Anaerobic toxicity tests (ATA) showed that acetic acid was completely converted to methane at 0.5% and 1% of fresh MWF, but bioconversion ‘lagged’ by approximately 7 and 15 days respectively. However, at 2% fresh MWF did not show any significant generation over a period of 175 days (Teli *et al*., 2015). In the same study, Teli *et al*. (2015) found very low biodegradation of fresh MWF in submerged anaerobic membrane bioreactors. In this study, the mechanisms for COD removal included bio‐adhesion of the MWF to anaerobic biomass, whereas COD membrane rejection was not significant. Perez *et al*. (2006) used an upflow anaerobic fixed‐film reactor (UAFF) to treat cutting oil wastewaters. The initial feed composed of wine vinasses was subsequently reduced while the amount of cutting oil was increased until 100% of cutting oil wastewater was added in the feed. At an organic loading rate (OLR) of 16.7 kg COD m^−3^ day (at cutting oil 100%), COD removal efficiency was 85.8%. Despite this, a very low level of biogas was found (0.0013 m^3^ CH_4_ kg COD^−1^). The same group (Perez *et al*., 2007) used an anaerobic thermophilic fluidized bed reactor to treat cutting oil wastewater. Over an operating period of 92 days, at an OLR of 11.9–51.3 kgCOD m^−3^ day^−1^ they achieved 67.1% COD and 71.3% TOC removal; however, the volumetric rate of biogas formation was very difficult to assess due to the small amounts that were generated. Conversely, Rodriguez‐Verde *et al*. (2014) examined the anaerobic digestion of spent MWF by testing the BMP and found 25% methane production with a yield of 15.6 l CH_4_ per kg of spent MWF. They did, however, increase the methane production up to 39% when the spent MWF was mixed with pig manure. Using DGGE profiling of the bioreactors, they found that *Firmicutes*, mainly *Clostridiaceae*, appeared in all samples, regardless of the operational performance, and concluded that this population was not sensitive to operational changes. The dominant archaea in an anaerobic bioreactor treating MWF and pig manure were found to be *Methanosaeta* species.

Previous anaerobic bioreactor studies have largely used inocula of undefined microbial communities from activated or anaerobic sludge (Perez *et al*., 2006; Rodriguez‐Verde *et al*., 2014; Teli *et al*., 2015). However, the antimicrobial nature of MWFs is invariably too hostile for such inocula, and especially to methanogens as was found in BMP and ATA tests by Teli *et al*., 2015. Carefully, constructed consortia of MWF resistant anaerobic bacteria may improve the opportunity to establish treatment systems.

The aim of this study was to test a proof of concept; that it is possible to anaerobically isolate strains (bacteria and methanogens) from spent MWF and then to construct a consortium using a random partitions design (RPD) mesocosm experiment. Then, if it is possible that this consortium could serve as the staring culture for a fluidized bed reactor (FBR) treating MWF.

## Results

### Isolation of COD reducing bacteria

Spent metalworking fluids from UK engineering works were stored for a year in 69, 1 l polythene containers. The percentage composition of headspace gas was analysed for methane (CH_4_) and CO_2_. Methane levels ranged from undetected (59/69 containers) to 12.5%, and CO_2_ levels ranged from undetected (3/69 containers) to 5.4%. Using the CO_2_ and CH_4_ levels as a guide, 43 containers of waste MWF were sampled for the isolation of COD reducers and the isolation of methanogens. Both hydrolytic and fermentative bacteria were isolated and colony purified on our media (below) developed for the liquid and plate culture of COD reducers, and incorporating fresh MWF. Using 16S rRNA gene sequencing, we identified 99 bacterial strains belonging to 27 species in 13 genera. The 99 isolates are listed in Table S1, and their phylogenetic tree is presented in Figure S1. The most commonly isolated bacteria belonged to the genus *Clostridium* (*n *=* *45) followed by the genera *Trabulsiella* and *Citrobacter* (both *n *=* *16). *Clostridium sporogenes* was the dominant species with 28 isolates, followed by *Trabulsiella odontotermitis* with 16 isolates and *Citrobacter amalonaticus* and *Serratia marcescens* both with five isolates (Table S1).

### MWF COD reduction by the isolated strains

This research was designed to inform on the potential to create starter or additive cultures for industrial bioreactors from bacteria pre‐adapted to toxic MWF environments. Strains were therefore selected primarily on their ability to reduce the MWF COD, but were excluded if there were valid health concerns. Specifically, strains were evaluated based on their pathogenicity based on the major public web databases in Germany, UK and USA. Purified strains were initially screened in liquid and solid media for rapid anaerobic growth with the challenge of 2% fresh MWF. A selection of 49 strains was then assayed for COD reduction over 28 days in liquid media with MWF (2% Castrol Cool Edge, initial COD of 12 000 mg COD l^−1^) as the sole carbon source (Table S2). Of these, 22 yielded COD reductions higher than 40%, whereas seven strains showed COD reduction lower than 10%. The strain *Clostridium metallolevans* B19 showed the highest COD reduction of 61.6%.

A subset of 16 strains was selected for strong COD reduction and growth, and as representative of the taxa identified. Where more than one isolate was selected from a species, phylogenetic data were used to avoid overly similar isolates being chosen. Please see supporting information regarding the detail procedure for selecting the 16 strains. Each MWF contains different chemical composition so the 16 isolates were also evaluated on a second MWF and their COD reduction on both MWFs is given in Table 1.

**Table 1:** COD reduction achieved by 16 bacterial strains on two freshly made metalworking fluids as sole carbon sources. Initial COD values were 14 500 mg COD l^−1^ for Castrol Oxford metalworking fluid (MWF) and 12 000 mg COD l^−1^ for Castrol Cooledge MWF

Strain code	Name (nearest match)	Oxford MWF COD reduction (%)	Cool Edge MWF COD reduction (%)	Included in mesocosms
B26	*Clostridium sporogenes*	49.7	33	Yes
B30	*Clostridium sporogenes*	44.7	38	Yes
B34	*Clostridium sporogenes*	23.1	48	Yes
D22	*Clostridium celerecrescens*	29.7	34	Yes
D46	*Clostridium mesophilum*	51.1	35	Yes
D45	*Clostridium propionicum*	37.9	33	Yes
B29	*Clostridium* sp.	61.1	36	No
C14	*Clostridium sulfidigenes*	49.8	38	Yes
B19	*Clostridium metallolevans*	61.6	42	Yes
B3	*Trabulsiella odontotermitis*	7.8	43	Yes
B8	*Trabulsiella odontotermitis*	4.8	47	Yes
B38	*Dethiosulfovibrio* sp.	27.4	33	No
C2	*Paenibacillus* sp. R2	32.4	33	No
D35	*Clostridium sartagoforme*	41.9	41	No
C11	*Serratia marcescens*	45.8	45	Yes
C21	*Sporanaerobacter* sp.	22.77	33	Yes

### Isolation of methanogens

Methanogens were isolated by the same approach as used to isolate the bacterial strains.

They were isolated and colony purified on our media (please see *Experimental Procedures* section) developed for the liquid and plate culture of methanogens, and incorporating fresh MWF. From a collection of 28 isolates, seven strains were found to grow rapidly (2–3 days) on acetic acid and formic acid with MWF, producing substantial CH_4_ (20–30% v/v) and were relatively easily re‐cultivated. Acetate is utilized by acetoclastic methanogens, whereas formic acid can be utilized by many hydrogenotrophic methanogens (Demirel and Scherer, 2008). These seven isolates were all identified as distinct strains of *Methanosarcina mazei*. The remaining 21 cultures showed slower growth (2 weeks) and low methane production so were excluded from further study. The seven *M. mazei* strains were further tested to confirm methane production in serum bottles with our methanogen selective broth and fresh MWF 0.5%. The 16S rRNA gene sequences were used to sub‐type these *M. mazei* strains and were designated M1, M2 and M3; their methane % gas composition production was 18.4%, 4.6% and 23.8%, respectively, growing for 28 days with acetic acid and formic acid as methanogenic carbon sources and with 0.5% Castrol Cooledge MWF to confirm tolerance. The three methanogens were also cultivated as a consortium which gave higher (additive) methane yields between 46% and 59%. This work shows that it is possible to isolate methanogenic strains, which are adapted to survival and function in MWFs, from industrial environments.

### Effects of bacterial diversity on the reduction in MWF COD

From the 16 bacteria listed in Table 1, 12 strains from nine species and four genera were selected on their ability to reduce the COD and to be representative of those taxa identified (also Table 1). This experiment was conducted with 12 species rather than 16 because in a random partition design (RPD) a 12 species design entails six levels of diversity (12, 6, 4, 3, 2 and 1 species), whereas a 16 species design has only five levels of diversity (16, 8, 4, 2 and 1 species; Bell *et al*., 2009).

**Figure 1:**
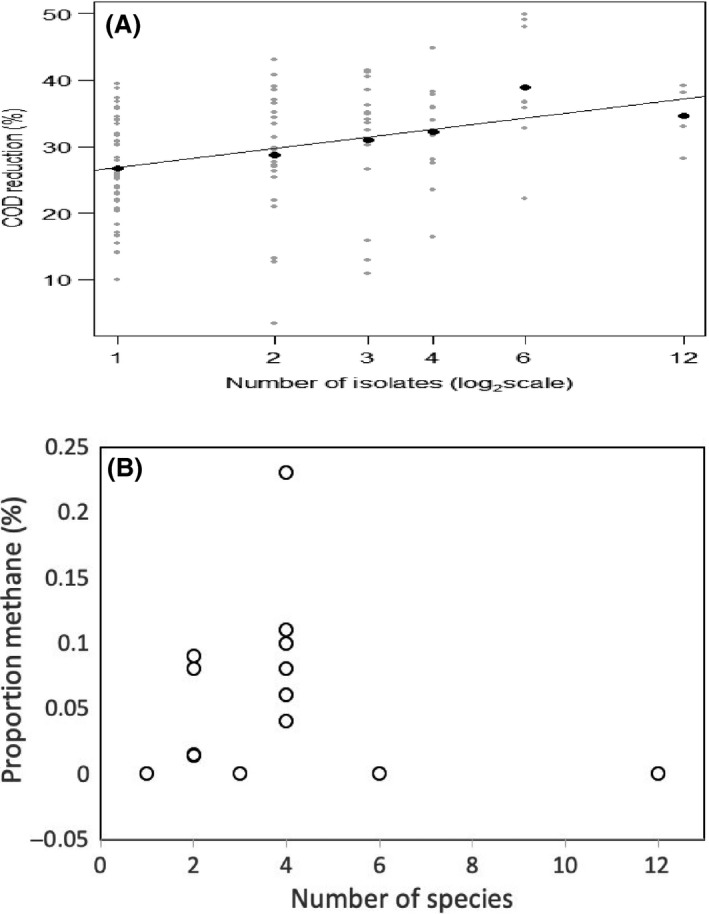
(a) COD reduction and (b) methane production plotted against number of species in the random partitions design experiment. Each microcosm is given its own point (•) with means taken across the microcosms with the same number of species present (•).

Bacteria were mixed anaerobically in various combinations (below) in 224 microcosms (serum bottles), each additionally with all three *Methanosarcina* mazei (Table 2). After 28 days, the reductions in COD varied between 3% and 52% (Fig. 1A). The statistical analysis of a general linear model has shown that: (i) there is a significant (*F*_1,18_ **=* *9.72, *P *=* *0.005) increase in COD reduction (3.15% COD per additional isolate) with increasing diversity (log_2_ transformed), (ii) the composition of species did not significantly (*F*_11,75_ **=* *0.94, *P *=* *0.511) affect COD reduction; however, (iii) there were significant effects of species–species interactions (*F*_4,18_ = 20.31, *P *<* *0.001).

**Table 2:** Methane yields (% headspace by volume) achieved by three methanogenic archaeal strains chosen to evaluate a metalworking fluid (MWF) treatment consortium after 28 days. The methane composition is from cultures with 0.5% Castrol Cooledge MWF to confirm tolerance and with acetic acid and formic acid as methanogenic carbon sources

Strain code	Name	Methane composition (%)
M1	*Methanosarcina mazei*	18.45
M2	*Methanosarcina mazei*	4.63
M3	*Methanosarcina mazei*	23.88

### Effects of bacterial diversity on methanogenisis

The 224 microcosms in the diversity‐RDP experiment each included the three *Methanosarcina* mazei strains (Table 2) and were assayed for the production of methane. The methane generated varied between 0% and 0.17% in the headspace gas composition. The relationship between methane production and the number of bacterial species present is shown in Fig. 1B (the samples generated 0% CH_4_ are not shown in the graph). In contrast with the COD reduction, it is clear that methane production peaks at four species. These results and the statistical analysis of a general linear model have shown that: (i) methane production increases with increasing diversity up to four species, (ii) the composition of bacterial species present in a mixture significantly affects the ability of the methanogens to generate methane, (iii) that there significant positive and negative species–species interactions affecting methane generation and (iv) there was a strong relationship between the reduction in COD and the production of methane *r*_(110)_ **=* *0.924, *P *=* *0.009.

Positive effects on methane production were noted in declining order from *Sporanaerobacter* sp. C21, *Dethiosulfovibrio* sp. B38, *Clostridium metallolevans* B19, *Clostridium* sp. B29 and *Clostridium sporogenes* B30. Negative effects were noted in declining order from *Clostridium mesophilum* D46, *Trabulsiella odontotermitis* B3, *Serratia marcescens* C11 and *Clostridium sporogenes* B26. However, significant interactions were noted and methane production is probably best associated with pairings of *Clostridium celerecrescens* D22, *Dethiosulfovibrio* sp. B38, *Paenibacillus* sp. C2 and *Serratia marcescens* C11, while *Trabulsiella odontotermitis* B3 appears to have been associated with loss of methane production. Other bacteria in mixtures appear to be impacting negatively on methane while one association (*Clostridium sporogenes B30*,* Clostridium* sp. B29, *Clostridium metallolevans* B19 and *Sporanaerobacter* sp. C21) is methane positive when the component parts would not predict this interaction.

### Operation of a fluidized bed bioreactor (FBR)

**Figure 2:**
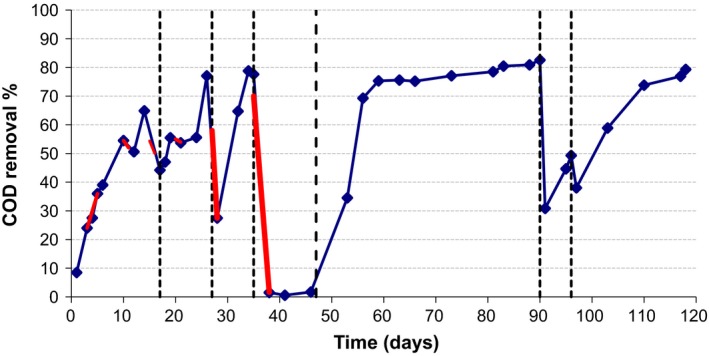
Plot of the removal of COD from metalworking fluid in fluidized bed bioreactor by the 19 strains listed in Tables 1 and 2.

Due to their being a significant increase in COD reduction with increasing diversity, a microbial consortium consisting of all 16 bacterial isolates (Table 1) and three methanogens were inoculated to initiate the FBR. To avoid the risk of washout of slow‐growing isolated strains, the FBR was alternated between continuous feed (48 h HRT) and batch mode (Table 3 reports information regarding the operation of FBR, feed composition and COD values). The COD removal periodically declined and was compensated for with: (i) the introduction of glucose, (ii) increased use of batch operation and (iii) the re‐introduction of the bacterial strains (Table 3). The FBR was started with 0.25% fresh MWF (1500 mg COD l^−1^), and then, the FBR was operated between batch mode and 48 h HRT with 0.5% fresh MWF (1–27 days). On day 34 under batch mode, the FBR exhibited abilities with nearly 80% COD removal (Fig. 2); however, when the FBR was shifted to 48 h HRT on day 35 the reactor was not stable and the COD removal was almost undetectable on day 46. On day 47, the FBR was operated under batch mode and this caused the COD removal to gradually increase from 1% (day 47) to 82.5% (day 91). An addition of 0.5% fresh MWF on day 97 resulted in the reduction of the COD removal to 50%. The addition of 1 g COD glucose l^−1^ as a co‐substrate on day 97 resulted in an increase performance of the bioreactor to 80% COD removal under batch mode (day 118; Table 3).

**Table 3:** The operational strategy for the operation of the fluidized bed bioreactor (FBR) bioreactor

Days	Operational mode	Feed addition
1–2	Batch	Feed with 0.25% fresh MWF + organic substrate (2890 mg COD l^−1^) = (3451 mg COD l^−1^)
3–5	48 HRT	
6–9	Batch	
10–11	48 HRT	
12–14	Batch	
15–16	48 h	Feed with 0.25% fresh MWF + organic substrate (1263 mg COD l^−1^) = 1824 mg COD l^−1^
17–19	Batch	
20–21	48 h	Addition of more Methanogens and COD reducers
22–26	Batch	
27–28	48HRT	Feed with 0.5% fresh MWF + organic substrate (1250 mg COD l^−1^) = 3310 mg COD l^−1^
29–34	Batch	
35–46	48 HRT	Batch Feed with 0.5% fresh MWF = 2650 mg COD l^−1^ (in the FBR)
47–91	Batch	No Feed addition
92–97	Batch	Batch Feed with 0.5% fresh MWF
98–118	Batch	Addition of Glucose as a co‐substrate 1000 mg COD l^−1^

**Figure 3:**
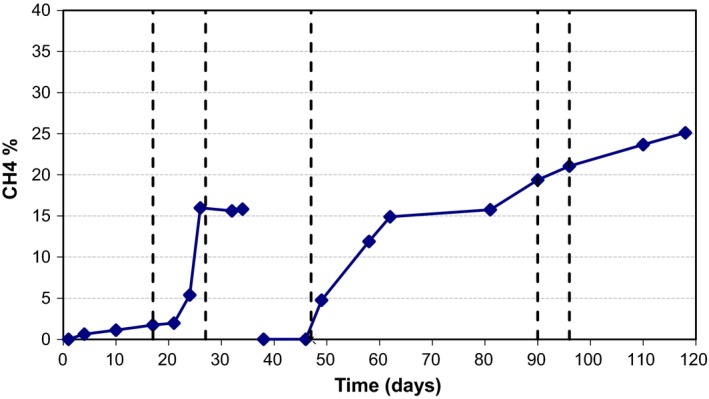
Methane composition in biogas over time from fluidized bed bioreactor (FBR) treating fresh metalworking fluid. During the 119 day FBR operation, the total volume of biogas produced was 850 ml.

The CH_4_ composition slowly increased during the operation to 15% on day 34. Based on this, it is pointed out that the majority of the COD is converted to CO_2_ (and not CH_4_), and therefore, during the FBR operation the anaerobic bacteria were the main COD utilizers. Then, the production of methane became variable falling to 0.5%. Gradually, CH_4_ increased to 25% on day 118 (Fig. 3). During the day 119 of FBR operation, the total volume of gas produced was 850 ml. However, CH_4_ composition never exceeded 26% during the FBR operation and taking in consideration the total generated gas; it is pointed out that the COD removal due to methanogenesis was around 15%. The rest of the amount of COD was converted to CO_2_ by anaerobic bacteria. The volatile fatty acids (VFAs) generated in the FBR were assayed. It was found that formic and acetic acid concentrations were 0–190 mg l^−1^ while n‐butyric acid concentrations were 380–610 mg l^−1^. Given the levels of methane generated, it was apparent that there were cessations in the reduction in the n‐butyric acid to substrates required for methanogenesis.

### Serum bottle experiments

**Figure 4:**
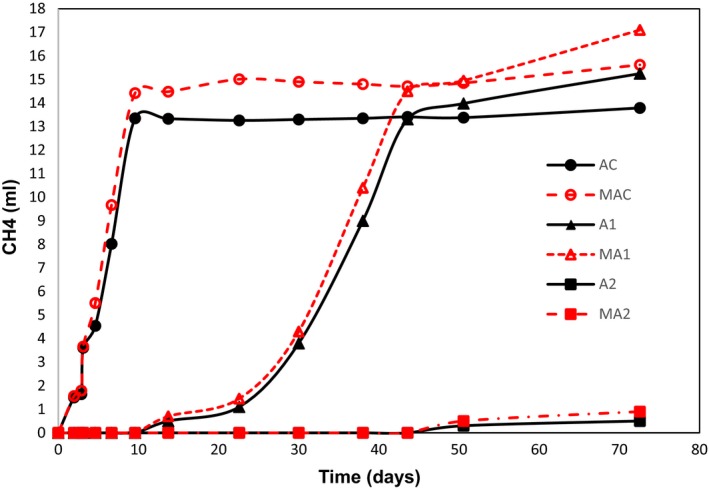
Methane production by anaerobic biomass (solid lines) and the anaerobic biomass + methanogens (dashed lines) in serum bottles. The anaerobic biomass was taken from an established continuous stirred‐tank reactor running on glucose, and the methanogenic strains were the three listed in Table 2. Key: (AC = acetate); MAC = control = anaerobic biomass + methanogenic strains + 2 g COD AC l^−1^; AC = control = anaerobic biomass + 2 g COD AC l^−1^; MA1 = anaerobic biomass + methanogenic strains + 2 gCOD AC l^−1^ + 1% fresh metalworking fluid (MWF); A1 = anaerobic biomass + 2 gCOD AC l^−1^ + 1% fresh MWF; MA2 = anaerobic biomass + methanogenic strains + 2 gCOD AC l^−1^ + 2% fresh MWF; A2 = anaerobic biomass + methanogenic strains + 2 gCOD AC l^−1^ + 2% fresh MWF.

Given the mixed results with the FBR, it was resolved to test methane production by the three methanogens. This assay in serum bottles measured methane production by anaerobic biomass in a two factored design; first, with and without added methanogens and second, with 0%, 1% (7250 mg COD l^−1^) and 2% (14500 mg COD l^−1^) fresh MWF. The second factor aimed to establish whether the MWF was inhibiting methanogenesis. The anaerobic biomass was taken from an established continuous stirred‐tank reactor running on glucose. In the control samples (no MWF), the methanogen supplemented biomass (Fig. 4) generated more methane (74% conversion of COD to CH_4_) than the anaerobic biomass alone (65% conversion of COD to CH_4_). This was also the case after the addition of 1% MWF; however, this addition delayed the generation of methane by ~40 days. Thus, the inclusion of the isolated methanogens resulted in both conditions around 10% higher CH_4_ production. The addition of 2% MWF almost completely suppressed the methanogenesis (Fig. 4), and as a result, negligible amount of COD to CH_4_ took place (around 2–3%). The results show that even in the presence of acetate, the fresh MWF severely inhibited methanogenesis by the anaerobic biomass, and that CH_4_ can be generated even in the presence of 2% fresh MWF after 70 days. In addition, we observed the production of methane (0.4% at 145 days) in 4% MWF when supplemented by acetate (data not shown), but not in the anaerobic biomass control, showing the methanogens are themselves not fatally inhibited by these concentrations of MWFs.

In other serum bottle assays run for 145 days with fresh MWF and no acetate, it became clear that including the 16 COD reducing strains and the three methanogenic strains, significantly increased the production of methane when MWF was 1% but not when 2% (data not shown). At the end of the experiment, 1% formic and acetic acid concentrations were up to 55 mg l^−1^ while n‐butyric acid concentrations were 440–510 mg l^−1^.

## Discussion

This study was designed to evaluate whether targeted isolation approaches could assemble a novel consortia of bacteria with the potential to initiate or supplement a bioreactor and facilitate COD reduction and methanogenesis in the challenging conditions of biocidal spent MWFs. The formation of the initial consortia was based on the following: (i) sampling from an appropriate (industrial) habitat, (ii) the culture of strains which are individually able, in the presence of a toxic MWF challenge, to either reduce MWF COD or generate methane and (iii) identification of these strains and then the selection of stains which are suitably active and representative of the taxonomic diversity observed. This ‘proof of concept’ study confirmed our approach as a viable response to this recalcitrant problem. A key finding is that it is possible, in industrial locations, to isolate methanogens which are adapted to survival and function in the hostile environment of MWFs. It was shown that the inoculating consortium could reduce COD (mainly to CO_2_), but could generate a modest amount of methane despite such challenges. This approach offers an alternative to the common practice of trying to adapt existing anaerobic bioreactor communities to spent MWFs or other biocidal substrates.

Reductions in COD and methanogenesis were significantly related to diversity and species–species interactions, though the methanogenesis–diversity relationship was weaker. COD reduction and methanogenesis were, respectively, non‐significantly and significantly related to composition. The effects of bacterial composition on archaeal methanogenesis are probably, in part, related to the varied performance of bacteria in reducing n‐butyric acid to acetate necessary for producing methane. However, our results indicated that bacterial interactions, rather than composition, were a significant factor in COD reduction. This suggests that the sum of the bacteria are more effective in reducing COD than the individual bacteria (Fiegna *et al*., 2015). Due to the time frames of the experiment, we postulate that bacteria may have started to utilize secondary metabolites (Lawrence *et al*., 2012). Previous work using bacterial consortia has demonstrated that bacteria can reduce potential antagonistic interactions through time (Rivett *et al*., 2016). This result is highly pertinent when assessing the competence of microbial consortia for industrial processes and thus merits further investigation. This reduction was strongly related to the production of methane. However, increasing the diversity of *M. mazei* from 1 to 3 strains resulted in a doubled methane yield. In the case of serum bottle experiments, the inclusion of the three isolated methanogens in mixed culture for fresh MWF biodegradation resulted in around 10% higher CH_4_ production compare with the anaerobic mix culture. At 1% fresh MWF, added acetic acid can be biodegraded although a lag phase of around 13 days was identified. Under similar conditions in the presence of 2% fresh MWF, methanogenesis was severely inhibited.

Formic and acetic acid are fermentative products from anaerobic bacteria which are substrates for methanogenesis. The periodic failure of the bacteria in the FBR to further reduce the n‐butyric acid to acetic acid and/or formic acid or to reduce the initial substrates to acetic acid and/or formic acid required for methanogenesis may explain some of the variability in our results (both in FBR and in serum bottles). This may be related to the dominant presence of *Clostridium* species, as many species produce butyric acid as an end‐product from substrate fermentation. If so, the solution may be to supplement with acidogenic bacteria capable of biodegrading butyric acid to acetic acid or the inclusion of anaerobic strains that can biodegrade MWF to acetic acid. However, we have confirmed that all the bacterial strains chosen are capable of COD reduction in CO_2_ in these conditions. It is possible that the problems in the FBR arose from variable expression of enzymes for the reduction in n‐butyric products. Further, the experiment testing diversity effects indicate that we may have included some bacteria with the potential for negative interactions reducing COD removal and production of methane.

The FBR exhibited limited progress in the difficult task of anaerobic treatment of fresh MWFs with biogas production. It would be useful to evaluate the effect of the constructed consortium (i) by bioaugmentation of the consortium to an anaerobic sludge. Some of the limitations of the existing study (e.g. accumulation of butyric acid) may be overcome by the synergistic effects of other microbes that already exist in anaerobic sludge. (ii) Another option is the consortium to be used as a starting FBR inoculum fed with fresh MWF; this could create the opportunity for further adaptation of the bioreactor community and then by sampling and incorporation of other suitably adapted bacteria.

*Clostridium* was the most common genus found with 45 of the 99 isolates identified. *Clostridium* spp. has the capacity to sporulate when environmental conditions become hostile, for example during heat shock, changes in nutrients status, presence of deleterious chemicals (Kapdan and Kargi, 2006); as the spores are metabolically dormant they are resistant to these stressors. This fact has been used by several authors to eliminate or kill non‐spore‐forming microorganisms, mainly methanogens, by means of a heat treatment. Investigations on microbial diversity of a mesophilic hydrogen producing sludge indicated the presence of Clostridia species as 64.6% (Kapdan and Kargi, 2006). Although *Clostridium* species have been found previously in MWF (Baker *et al*., 1983; Gilbert *et al*., 2010), no other study has reported the *Clostridium* strains found in this study.

Industrial machines and equipment operate in high temperatures with exposures above > 80°C and therefore select for certain species that can survive under these fluctuating conditions. *Citrobacter* species were accounted for 16 of the 99 isolates. Van der Gast *et al*. (2001) found that *Citrobacter freundii* accounted for 19%, of all isolates (aerobic) that were isolated from used MWF. *Citrobacter freundii* causes occasional human infections, but is also known to produce H_2_S and organic acids during its anaerobic metabolism (Borenshtein and Schauer, 2006), suggesting that its presence could significantly lower MWF pH and participate in toxic gas production in anaerobic conditions. Further, Gilbert *et al*. (2010), Lonon *et al*. (1999) and Mattsby‐Baltzer *et al*. (1989) identified the *Citrobacter freundii* in used MWF. In our study, *C*. *freundii* accounted for 16%, of all anaerobic/facultative bacteria isolated from used MWF. LeChevallier *et al*. (1980) and Payment (1989) isolated *C. freundii* from distribution water and from raw water. It is possible during dilution of fresh MWF with water that MWFs will be contaminated with microorganisms found in raw water. Furthermore, Mattsby‐Baltzer *et al*., 1989, and Gilbert *et al*., 2010, also identified the *Citrobacter diversus* and *Citrobacter farmer,* respectively, from a spent MWF. However, no other study has reported the citrobacter strains: *amalonaticus, murliniae*, sp. *SR3* and sp. *F1‐1* that were found in this study.

*Methanosarcina mazei* are of great ecological importance as they are the only organisms known to ferment acetate, methylamines and methanol to methane, carbon dioxide and ammonia. It is likely that the methanogens present in used MWF are originally from soil. However, given their atypical resistance to MWFs and their diversity, it is probable that these strains have a history of adaptation in engineering systems. The findings of this study are in line with a review (De Vrieze *et al*., 2012) which reported that *Methanosarcina* sp. are tolerant to high levels of ammonium, salt and acetate and in general can be found in harsh conditions. Interestingly, Di Maiuta *et al*. (2017) used parallel ribosomal gene tag sequencing and found Archaea in two of 78 samples analysed. All reads were classified into the genus of *Methanobrevibacter* spp. with an origin from human sources considered likely as such.

The FBR results illustrate the challenges of biogas production by a designed microbial consortium. Despite these, the FBR was operated under batch condition with methane composition below 40%. Teli *et al*. found low biodegradation of MWF in SAnMBR inoculated with anaerobic sludge. On the other hand, several studies used the biological processes along with other physicochemical processes and achieved higher overall performance. Specifically, Jagadevan *et al*., 2013 used a hybrid ozone‐bacteriological treatment and achieved 72% COD removal from MWF. Thill *et al*. (2016) used biological, nanoscale zero‐valent iron and electron beam irradiation treatment and reduced the MWF COD by 92.8%. The results of the current study indicate that another process after or prior anaerobic treatment could be used for further COD reduction.

## Experimental procedures

### Procedure for isolation and purification of COD reducers strain and methanogens from used MWF

Waste MWF from a variety of sources was provided by Microbial Solutions Limited in plastic containers where they had been sealed and stored (ambient temperature) for about a year. Sixty‐nine of these containers were selected for consideration, and their headspace gas composition analysed. Samples were taken from these containers and were inoculated in 165‐ml serum bottles with a working volume of 100 ml. The working volume of 100 ml included the following: (i) spent MWF (inoculum) = 20 ml, (ii) fresh MWF at 0.5% to 2% v/v (the COD values for fresh MWF were 14500 mg COD l^−1^ for Castrol Oxford MWF and 12 000 mg COD l^−1^ for Castrol Cooledge MWF), (iii) 1 ml (1% v/v) of stock solution, containing‐ K_2_HPO_4_ (3 g l^−1^), NaCL (2 g l^−1^), Na_2_CO_3_ (2 g l^−1^), (NH_4_)2PO_4_ (2 g l^−1^), L‐Cysteine (0.5 g l^−1^), CaCL_2_.2H2O (0.2 g l^−1^), MgCL_2_.6H_2_O (0.2 g l^−1^). Trace elements stock solution contained the following: MgSO_4._7H_2_O (3 g l^−1^), MnSO_4._H_2_O (0.5 g l^−1^) NaCL (1 g l^−1^), FeSO_4._7H_2_O (0.1 g l^−1^), CoCL_2_.6H_2_O (0.1 g l^−1^), CaCL_2_ (0.1 g l^−1^), ZnSO_4._7H_2_O (0.1 g l^−1^), CuSO_4._7H_2_O (10 mg l^−1^), ALK(SO_4_)_2_.12H_2_O (10 mg l^−1^), H_3_BO_3_ (10 mg l^−1^), Na_2_MoO_4_ (10 mg l^−1^), NiSO_4._6H_2_O (30 mg l^−1^), Na_2_SeO_3_ (20 mg l^−1^), Na_2_WO_4_.2H_2_O (20 mg l^−1^), nitrilotriacetic acid C_6_H_9_NO_6_ (1.5 g l^−1^). Media for liquid and plate culturing were designed by reviewing a range of standard practices and adapting them to our aims. The media were flushed with 70% N_2_ and 30% CO_2_ for 15 min. The pH was adjusted to 7–7.3 with 50% HCl. The serum bottles were flushed with 70% N_2_ and 30% CO_2_ for 3 min and then sealed under anaerobic conditions. COD reduction and methane production were monitored, and the liquid cultures were then spread on plates and to isolate and purify strains. The solid media contained the following: peptone 10 (g l^−1^), yeast extract (5 g l^−1^), K_2_HPO_4_ (3 g l^−1^), NaCl (2 g l^−1^), Na_2_CO_3_ (2 g l^−1^), (NH_4_)_2_PO_4_ (2 g l^−1^), l‐Cysteine (0.5 g l^−1^), CaCL_2._2H_2_O (0.2 g l^−1^), MgCL_2._6H_2_O (0.2 g l^−1^), agar technical (16 g l^−1^) and trace elements 1% v/v. The pH was adjusted to 7–7.3 using HCl (50%). The plates were incubated at 37°C in 3.5‐l anaerobic jars (Oxoid). Anaerobic conditions were achieved by inclusion of anaerobic gas kits (AnaeroGen Oxoid, Oxoid Limited, Basingstoke, UK).

The serum bottle experiment with addition of acetic acid and fresh MWF was taken place at a working volume of 30 ml in a 50‐ml serum bottle.

### Procedure for isolation, purification and identification of methanogens

A portion of 5 ml from the each spent MWF, where CH_4_ was detected, was placed in serum bottles (40 ml), with autoclaved media (15 ml) which contained the following: NaOH (2 g l^−1^), yeast extract (2 g l^−1^), peptone (2 g l^−1^), NH_4_Cl (1 g l^−1^), K_2_HPO4.3H_2_O (0.4 g l^−1^), MgCL2.6H2O (1 g l^−1^), CaCl_2_.2H2O, resazurin (1 mg l^−1^), Na_2_S.9H_2_O (0.25 g l^−1^), mercaptoethanesulfonic acid (0.5 g l^−1^) and trace elements 1% v/v as in the procedure above. Acetic acid (1.5 g l^−1^) and formic acid (0.5 g l^−1^) were added. The media was flushed for 15 min with 70% N_2_ and 30% CO_2_. The pH was adjusted to 7–7.3 using HCL (50%). Then, the medium in the serum bottle was flashed with 70% N_2_ and 30% CO_2_ for 3 min. Each used MWF was subjected to 0%, 0.5%, 1% and 2% fresh MWF. After 1–2 months, liquid samples were taken from the serum bottles that had more than 5% CH_4_ and spread for colony purification on plates made with an autoclaved medium containing NaOH (2 g l^−1^), yeast extract (1 g l^−1^), peptone (1 g l^−1^), NH4Cl (1 g l^−1^), K_2_HPO_4_.3H_2_O (0.4 g l^−1^), MgCL_2_.6H_2_O (1 g l^−1^), CaCL_2_.2H_2_O, Na_2_S.9H_2_O (0.25 g l^−1^), mercaptoethanesulfonic acid (0.5 g l^−1^), trace elements 1%v/v, acetic acid (1.5 g l^−1^), formic acid (0.5 g l^−1^) and agar technical (16 g l^−1^). The pH was adjusted to 7–7.3 using HCl (50%). The plates were incubated at 37°C in an anaerobic jar. Individual strains were then tested for methane production in serum bottles with the above media with and without fresh MWF 0.5%. Please see supporting information regarding procedure for DNA extraction, PCR and sequencing.

### Testing diversity effects

We tested novel combinations of 12 COD reducing strains in order to identify positive and negative synergies and more general effects of increasing diversity on the degradation of MWFs. These strains were ‘Partitioned’ in combinations (total *n *=* *28) of two spp. (*n *=* *6), three spp. (*n *=* *4), four spp. (*n *=* *3), six spp. (*n *=* *2), 12 spp. (*n *=* *1) and 1 spp. (*n *=* *12). The multiple species combinations were randomly assembled using the framework of the random partitions design (RDP; Bell *et al*., 2005, 2009), with each of the strains present once at each of the richness levels. These partitions of species combinations were independently randomly re‐assembled four times. All microcosms were replicated twice (*n *=* *224).

The pre‐assembled bacterial combinations were used to inoculate 50‐ml serum bottles containing 0.5% MWF 30 ml (3000 mg COD l^−1^) as the sole carbon source together with three, pre‐inoculated, strains of the methanogenic archaea *Methanosarcina mazei*. The microcosms were incubated anaerobically at 28°C for 28 days, and after which, the reduction in COD and methane production were assayed as described in *Analytical Techniques* section.

This design facilitated a statistical analysis (Bell *et al*., 2005) that differentiated between the influences of the following: (i) diversity *per se* (number of strains), (ii) individual strains (composition) and (iii) interactions between strains.

### Analytical techniques

Mixed liquor total and volatile suspend solids (MLSS, MLVSS, COV = ± 5%) and COD (COV = ± 5%) were measured according to the standard methods for the examination of water and wastewater (APHA, 2005). VFAs were measured on Shimadzu HPLC using a Biorad‐Aminex column, and the carrier solvent was 0.01 M H_2_SO_4_ at a flow rate of 0.7 ml min^−1^ at 60°C (COV = ± 8%). The composition of biogas was determined using a Shimadzu GC‐TCD fitted with a Porapak N column (1500 × 6.35 mm; Vyrides, 2009).

### Fluidized bed bioreactor

A FBR and associated equipment were kindly provided by Dr. Paul Sallis at Newcastle University. The FBR reactor had a total volume of 8.5 l, a working volume of 6.5 l and was kept at 37°C. A key objective in establishing the FBR reactor was the retention of the selected strains inside the bioreactor. Powdered activated carbon (PAC) was added (2 g l^−1^) to provide surface area for bacterial adsorption and growth. The properties of the PAC are given in Table S3.

## Conflict of interest

The authors declare that there are no conflicts of interest.

## Supporting information


**Appendix S1.** Rational criteria for the selection of microbial consortia.
**Appendix S2.** Methods‐DNA extraction and PCR
**Table S1.** Summary of the 99 strains isolated from waste MWFs.
**Table S2.** Results of COD reduction assays for 37 strains.
**Table S3.** Characteristics of the Norit Powdered Activated Carbon SAE‐2.
**Fig. S1.** A phylogenetic tree showing relationships between the 99 strains isolated from waste MWFs. The tree was calculated using 16S rRNA gene partial sequences and the Ribosomal Database Project facility.
