# Supplementary material for: Selection and assembly of indigenous bacteria and methanogens from spent metalworking fluids and their potential as a starting culture in a fluidized bed reactor
Source: Microb Biotechnol. 2019 Jul 21;12(6):1302–12. doi: 10.1111/1751-7915.13448 (PMC6801153; doi:10.1111/1751-7915.13448)
Supplement: Supplementary file 1 — Appendix S1. Rational criteria for the selection of microbial consortia. Appendix S2. Methods‐DNA extraction and PCR Table S1. Summary of the 99 strains isolated from waste MWFs. Table S2. Results of COD reduction assays for 37 strains. Table S3. Characteristics of the Norit Powdered Activated Carbon SAE‐2. Fig. S1. A phylogenetic tree showing relationships between the 99 strains isolated from waste MWFs. The tree was calculated using 16S rRNA gene partial sequences and the Ribosomal Database Project facility. [file MBT2-12-1302-s001.docx]

Supporting Information

**Selection and assembly of indigenous bacteria and methanogens from spent Metal Working Fluids and their potential as a staring culture in a Fluidized Bed Reactor**

Vyrides I^1,2^, Rivett, D.W^1,3^, Bruce, K.D^1^. Lilley, A.K^1^*

1: King's College London, Institute of Pharmaceutical Sciences, 150 Stamford Street, Franklin-Wilkins Building, London, SE1 9NH, UK

2: Department of Environmental Science and Technology, Cyprus University of Technology, 30 Archbishop Kyprianos, 3036 Lemesos, Cyprus.

3: Division of Ecology and Evolution, Imperial College London, Silwood Park Campus, Ascot, Berkshire SL5 7PY, UK.

Corresponding author: [andy.lilley@kcl.ac.uk](mailto:andy.lilley@kcl.ac.uk)

**Contents**

- **Rational criteria for the selection of microbial consortia.**

-**Methods**

-**DNA extraction and PCR**

- **Table S1.** Summary of the 99 strains isolated from waste MWFs.

- **Table S2.** **Results of COD reduction assays for 37 strains.** The assays were made over 28 days using Castrol Cool Edge MWF at 2% concentration giving an initial COD of 12000 mgCOD/L.

- **Table S3.** Characteristics of the Norit Powdered Activated Carbon SAE-2.

- **Figure S1.** A phylogenetic tree showing relationships between the 99 strains isolated from waste MWFs. The tree was calculated using 16S rRNA gene partial sequences and the Ribosomal Database Project facility.

**Rational criteria for the selection of strains for the microbial consortia.**

A panel of 16 bacteria (Table 1) was constructed for consortia evaluation in diversity-function studies (n = 12) and as an inoculum in a fluidized bed bioreactor (FBR) (n = 16). In addition 3 methanogens were included to evaluate the potential for methane production.

These 16 were chosen on the following criteria: a) Selection of the most common taxa

(*Clostridium*) that showed the highest COD removal (Table 1). *Clostridium sporogenes* was the most commonly observed species in waste MWF despite that is not a pathogen is closely related to *Clostridium botulinum*, a serious health hazard. For this reason *Clostridium sporogenes* were tested, and found negative, for the four botulinum toxins hazardous to humans

b) In some cases more than one strain was chosen from a genera or species such as *Clostridium sporogenes* and *Trabulsiella odontotermitis*. Phylogenetic trees were constructed to ensure that genetically distinct strains were chosen. This avoided the inclusion of two overly similar strains.

c) Selection of genus that were not abandoned among the isolated 99 and pointed out independent COD removal such as *Dethiosulfovibrio, Paenibacillus* . These strains can sustain the stability of the consortium in environmental variations.

d) To avoid the inclusion of strains which are pathogens, or which have identities which imply risk. All the 99 strains were evaluated using three major public web data bases in Germany, UK and USA. The method sought bacteria with a classification equivalent to the UK's 'Hazard Group 1', which has been defined by the Advisory Committee on Dangerous Pathogens (ACDP) as "a biological agent unlikely to cause human disease". It **cannot** be assumed, that these bacteria, or related bacteria, will be innocuous in all situations or that infection can never occur. Using this approach a number of the 99 isolated strains were rejected from inclusion in waste MWF degrading consortia. These include all strains of *Citrobacter, Morganella, Proteus, Salmonella, Escherichia, Klebsiella, Tissierella* and poorly recognized (e.g. uncultured) strains.

**Methods- DNA extraction and PCR**

DNA extraction used a DNA isolation kit (MO BIO Ultra Clean DNA isolation Kit, NEED LOCATION) , PCR reaction was taken place using the primers PA: 5’ AGA GTT TGA TCC TGG CTC AG 3’ and pH: 5’-AAG GAG GTG ATC CAG CCG CA 3 ’ and the following program was used : 94 ^0^C for 2min and then 33 cycles consisted of 94 ^0^C for 1 min 56 ^0^C for 1min -72 ^0^C for 2 min and final step of 72 ^0^C for 7 min. For the methanogens PCR reaction was taken place using the primers 21F (5’ TTCCGGTTGATCCYGCCGGA-3’ and 958R (5’YCCGGCGTTGAMTCCAATT-30 and the following program was used : 95^o^C for 5min and then 30 cycles consisted of 94 ^o^C for 1 min 55 ^0^C for 1min -72 ^0^C for 1 min and final step of 72 ^0^C for 10 min. Then the samples were purified by PCR purification kits (MO Bio Ultra clean PCR purification Kit). Following sequencing (Macrogen, Nederlands) the following accession codes were obtained for the microbes in this study

**Table S1.** Summary of the 99 strains isolated from waste MWFs. Identifications made by 16S rRNA

| Identity base on the 16S rRNA gene nearest match | No by species | No by genus |
| --- | --- | --- |
|  |  |  |
| *Citrobacter* |  | 16 |
| *Citrobacter amalonaticus* | 5 |  |
| *Citrobacter freundii* | 4 |  |
| *Citrobacter murliniae* | 3 |  |
| *Citrobacter* sp. SR3 | 4 |  |
|  |  |  |
| *Clostridium* |  | 45 |
| *Clostridium celerecrescens* | 3 |  |
| *Clostridium mesophilum* | 3 |  |
| *Clostridium metallolevans* | 1 |  |
| *Clostridium propionicum* | 2 |  |
| *Clostridium sartagoforme* | 1 |  |
| *Clostridium sordellii* | 1 |  |
| *Clostridium* sp. D3RC-1 | 2 |  |
| *Clostridium* sp. U42 | 1 |  |
| *Clostridium sporogenes* | 28 |  |
| *Clostridium sulfidigenes* | 1 |  |
| *Clostridium thiosulfatireducens* | 2 |  |
|  |  |  |
| *Dethiosulfovibrio* sp. USBA 82 | 1 | 1 |
|  |  |  |
| *Escherichia coli* | 2 | 2 |
|  |  |  |
| *Klebsiella oxytoca* | 1 | 1 |
|  |  |  |
| *Morganella morganii* | 4 | 4 |
|  |  |  |
| *Paenibacillus* sp. R2 | 1 | 1 |
|  |  |  |
| *Proteus vulgaris* | 3 | 3 |
|  |  |  |
| *Serratia* |  | 6 |
| *Serratia* sp. xC2 | 1 |  |
| *Serratia* marcescens | 5 |  |
|  |  |  |
| *Sporanaerobacter acetigenes* | 1 | 1 |
|  |  |  |
| *Tissierella* sp. S2 | 1 | 1 |
|  |  |  |
| *Trabulsiella odontotermitis* | 16 | 16 |
|  |  |  |
| Uncultured Bacterium | 2 | 2 |
|  |  |  |
| N | 99 | 99 |

**Table S2.** Results of COD reduction assays for 49 strains. The assays were made over 28 days using Castrol Cool Edge MWF at 2% concentration giving an initial COD of 12000 mgCOD/L. 53

| Code | Species (nearest match) | COD reduction (%) |
| --- | --- | --- |
| B3 | *Trabulsiella odontotermitis* | 43 |
| B4 | *Citrobacter sp SR3* | 6 |
| B5 | *Citrobacter amalonaticus* | 5 |
| B8 | *Trabulsiella odontotermitis* | 47 |
| B13 | *Serratia marcescens* | 45 |
| B17 | *Clostridium sp D3RC-1* | 53 |
| B19 | *Clostridium metallolevans* | 42 |
| B20 | *Trabulsiella odontotermitis* | 56 |
| B24 | *Clostridium mesophilum* | 58 |
| B25 | *Citrobacter freundii* | 45 |
| B26 | *Clostridium sporogenes* | 33 |
| B28 | *Morganella morganii* | 35 |
| B29 | *Clostridium sp D3RC-1* | 36 |
| B30 | *Clostridium sporogenes* | 38 |
| B34 | *Clostridium sporogenes* | 48 |
| B38 | *Dethiosulfovibrio sp* | 33 |
| B40 | *Clostridium sordellii* | 52 |
| C2 | *Paenibacillus sp R2* | 33 |
| C8 | *Klebsiella oxytoca* | 18 |
| C10 | *Tissierella sp S2* | 13 |
| C11 | *Serratia marcescens* | 45 |
| C12 | *Citrobacter murliniae* | 26 |
| C13 | *Clostridium propionicum* | 17 |
| C14 | *Clostridium sulfidigenes* | 38 |
| C16 | *Serretia marcescens* | 7 |
| C20 | *Clostridium thiosulfatireducens* | 55 |
| C21 | *Sporanaerobacter* sp. | 33 |
| D2 | *Clostridium sporogenes* | 54 |
| D7 | *Uncultured Bacterium* | 23 |
| D15 | *Proteus vulgaris* | 51 |
| D16 | *Morganella morganii* | 7 |
| D19 | *Clostridium thiosulfatireducens* | 51 |
| D20 | *Citrobacter amalonaticus* | 47 |
| D22 | *Clostridium celerecrescens* | 34 |
| D30 | *Clostridium sp U42* | 15 |
| D33 | *Escherichia coli* | 14 |
| D35 | *Clostridium sartagoforme* | 41 |
| D37 | *Serratia sp xC2* | 8 |
| D38 | *Uncultured Bacterium* | 32 |
| D40 | *Serretia marcescens* | 10 |
| D41 | *Proteus vulgaris* | 58 |
| D42 | *Escherichia coli* | 17 |
| D43 | *Clostridium celerecrescens* | 45 |
| D45 | *Clostridium propionicum* | 33 |
| D46 | *Clostridium mesophilum* | 35 |
| D48 | *Clostridium celerecrescens* | 7 |
| D50 | *Citrobacter sp. F1-1* | 5 |
| D52 | *Morganella morganii* | 20 |
| D54 | *Citrobacter freundii* | 51 |

**Table S3.** Characteristics of the Norit Powdered Activated Carbon SAE-2

| Total surface area (m^2^/g) | 925 |
| --- | --- |
| Apparent density (kg/m^3^) | 450 |
| Porosity (%) | 55.7 |
| Pore size distribution (µm) | 90.61-0.0055 |
| Median pore diameter (µm) | 2.860 |
| Average Pore diameter(4V/A) (µm) | 0.148 |
| Particle size >150 µm (mass-%) | 3 |
| Particle size D_50_ µm | 22 |
| pH | alkaline |
| Moisture (max. mass %) | 6 |
| Iodine number (mg/g) | 850 |
| Methylene blue adsorption (g/100g) | 12 |

**
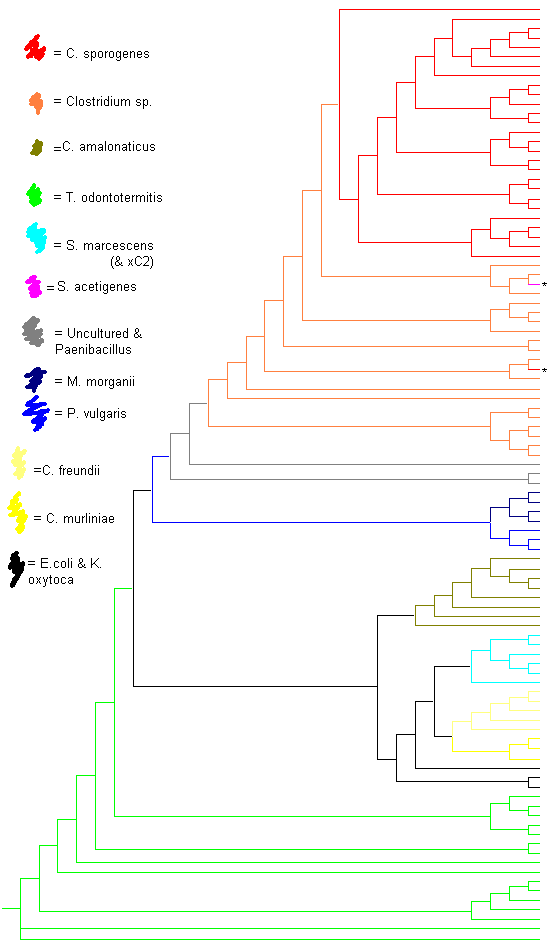
Figure S1.** A phylogenetic tree showing relationships between the 99 strains isolated from waste MWFs. The tree was calculated using 16S rRNA gene partial sequences and the Ribosomal Database Project facility.
